# Vascular Aging: Facts and Factors

**DOI:** 10.3389/fphys.2012.00325

**Published:** 2012-08-10

**Authors:** Ana P. Dantas, Francesc Jiménez-Altayó, Elisabet Vila

**Affiliations:** ^1^Institut Clínic del Tòrax, Institut d'Investigacions Biomèdiques August Pi i SunyerBarcelona, Spain; ^2^Departament de Farmacologia, Terapèutica i Toxicologia, Facultat de Medicina, Institut de Neurociències, Universitat Autònoma de BarcelonaBellaterra, Spain

“Man is as old as his arteries.” This old aphorism has been widely confirmed by epidemiological and observational studies establishing that cardiovascular diseases can be age-related in terms of their onset and progression. Besides, with aging come a number of physiological and morphological changes that alters cardiovascular function and lead to subsequently increased risk of cardiovascular disease, even in health asymptomatic individuals. Even though different adaptive mechanisms to protect blood vessels against mild stress have been described, the aging process induces a progressive failure of protective mechanisms, leading to vascular changes. The outcomes of the aging-related modifications are the impairment of homeostasis of the irrigated organs and resultant target organ damage.

The increasing mean age of the population in industrialized countries has turned out to be an economic and public health problem, as the increase in life expectancy goes in parallel with high incidence of several pathological conditions, despite unprecedented advances in prevention, diagnostics, and treatment. Of all aging-related illness, cardiovascular diseases remain the leading cause of morbidity and mortality in the elderly, and thus it is imperative to understand the mechanism underlying cardiovascular senescence.

This Research Topic presents a forum of comprehensive reviews on distinct aspects of the pathophysiology of vascular aging to provide insights into the causes and consequences of this complex process and attempts to propose new therapeutic strategies for the management of vascular senescence. Such as the idea proposed by Ming et al. (2012) and discussed by Cau and Tostes (2012), which suggests that targeting mTORC1-S6K1 signaling could be a promising therapeutic modality to retard the aging process and treat cardiovascular disease in the elderly.

Vascular aging could be simply described as a consequence of natural physical stress and fatigue that could account for the major physical changes seen in elderly: dilation (after fracture of load-bearing material) and stiffening (by transfer of stress to the more rigid collagenous component of the arterial wall). Although aging-associated changes on vascular functioning are considered a set up for cardiovascular disease, modifications on cardiac, and central function could slow down or accelerate this set point. Therefore, it is crucial to understand how aging and other pathophysiological states affect the interaction between the heart and arterial network. The article by Wojciechowska et al. (2012) provides information on the changes with age in central and peripheral systolic blood pressure, based on data collected from randomly recruited European and Chinese subjects supporting a vicious circle between age-related stiffness, increasing systolic blood pressure, and cardiovascular complications. Chantler and Lakatta (2012) elegantly describe the concept of arterial ventricular coupling and provide valuable information on how aging, in the absence and presence of cardiovascular disease, affects the coupling both at rest and during exercise, and its pathophysiological consequences.

In addition, vascular aging has been largely associated with senescence of the vascular endothelium. El Assar et al. (2012) present a wide overview of the mechanisms that participate on endothelial dysfunction that accompanies vascular aging, analyzing the synergisms, and interactions between them, some of the cellular mechanisms related to endothelial senescence as well as the prevention or reversion of those mechanisms that produce endothelial dysfunction. Cau et al. (2012) based on evidence from experimental models review the contribution of NO synthase (NOS) isoform alterations on aging-associated vascular dysfunctions, addressing the potential prevention by some drugs that modulate the expression/activity of NOS. Finally, Blanco and Bernabéu (2012) summarize data supporting the link between the splicing factor SRSF1 and endothelial cell senescence and suggest the existence of a common genetic program involving alternative splicing of a cluster of genes, which contributes to a senescent environment in the vessels. Nonetheless, aging-associated damage to the endothelium may not simply be a consequence of the endothelial cell malfunctioning, but also as a result of impaired maintenance repair systems by endothelial progenitor cell (EPC). As discussed by Williamson et al. (2012), a deterioration of endogenous EPC function with age may culminate in a decreased capacity for neovascularization and/or reduced re-endothelialization of vascular lesions, facilitating the development, progression, and clinical sequelae of cardiovascular disease.

Growing evidence show that the progress of vascular aging in women follows a different chronology than in men. The gender-associated difference in the pathophysiology of cardiovascular disease has generated heated discussion, although a general consensus has validated a role of sex hormones in the modulation of vascular function and dysfunction. This research topic covers two fronts in this field: Novella et al. (2012) review clinical and experimental data to clarify how menopause and aging contribute jointly to vascular senescence and how estrogen modulates vascular response at different ages; Lopes et al. (2012) discuss the conflicting information on the role of testosterone to the regulation of vascular function in elder men and women.

Last but not least, as suggested by Gragasin et al. (2012) the elderly is more frequently represented in common medical procedures and surgeries. Thus, understanding the circulatory changes that accompany the aging process is therefore becoming increasingly timely and relevant. These authors discuss aspects of vascular control in aging that are particularly relevant in the maintenance of intraoperative hemodynamic stability reviewing the effect of certain notable anesthetic agents with respect to the aging vasculature.

Given the growing clinical relevance of the subject, we are pleased that our Research Topic has brought together basic and clinical scientists to spotlight on one of the greatest enemies of elder population: i.e., the vascular senescence. We hope that this Research Topic also place a special call on the need of studies to establish treatments and procedures to reduce the detrimental effects of vascular aging. It has been a great pleasure to be involved in this Research Topic and we would like to thank all of the authors, reviewers, and Frontiers staff for helping to make this Research Topic possible.
